# Real-Time Multiphoton Intravital Microscopy of Drug Extravasation in Tumours during Acoustic Cluster Therapy

**DOI:** 10.3390/cells13040349

**Published:** 2024-02-16

**Authors:** Jessica Lage Fernandez, Sofie Snipstad, Astrid Bjørkøy, Catharina de Lange Davies

**Affiliations:** 1 Department of Physics, Norwegian University of Science and Technology, 7034 Trondheim, Norway; sofie.snipstad@ntnu.no (S.S.); astrid.bjorkoy@ntnu.no (A.B.); catharina.davies@ntnu.no (C.d.L.D.); 2 Cancer Clinic, St. Olavs Hospital, 7030 Trondheim, Norway

**Keywords:** intravital microscopy, microbubbles, drug delivery, extravasation, dorsal window chambers

## Abstract

Optimising drug delivery to tumours remains an obstacle to effective cancer treatment. A prerequisite for successful chemotherapy is that the drugs reach all tumour cells. The vascular network of tumours, extravasation across the capillary wall and penetration throughout the extracellular matrix limit the delivery of drugs. Ultrasound combined with microbubbles has been shown to improve the therapeutic response in preclinical and clinical studies. Most studies apply microbubbles designed as ultrasound contrast agents. Acoustic Cluster Therapy (ACT^®^) is a novel approach based on ultrasound-activated microbubbles, which have a diameter 5–10 times larger than regular contrast agent microbubbles. An advantage of using such large microbubbles is that they are in contact with a larger part of the capillary wall, and the oscillating microbubbles exert more effective biomechanical effects on the vessel wall. In accordance with this, ACT^®^ has shown promising therapeutic results in combination with various drugs and drug-loaded nanoparticles. Knowledge of the mechanism and behaviour of drugs and microbubbles is needed to optimise ACT^®^. Real-time intravital microscopy (IVM) is a useful tool for such studies. This paper presents the experimental setup design for visualising ACT^®^ microbubbles within the vasculature of tumours implanted in dorsal window (DW) chambers. It presents ultrasound setups, the integration and alignment of the ultrasound field with the optical system in live animal experiments, and the methodologies for visualisation and analysing the recordings. Dextran was used as a fluorescent marker to visualise the blood vessels and to trace drug extravasation and penetration into the extracellular matrix. The results reveal that the experimental setup successfully recorded the kinetics of extravasation and penetration distances into the extracellular matrix, offering a deeper understanding of ACT’s mechanisms and potential in localised drug delivery.

## 1. Introduction

A major problem in treating cancer is the low and heterogeneous uptake of drugs in tumour tissue and its toxic effects against normal tissue [1,2]. Several strategies to develop a tumour-specific accumulation of drugs have been proposed, such as nanomedicines, targeting ligands, and therapies for specific gene mutations [3]. However, the clinical effects are limited [4,5]. Drug delivery to tumours is affected by several biological barriers and processes that impede the access of the chemotherapeutic agents into the tumour, such as short circulation time, vascular barriers including passage across the capillary wall, high interstitial fluid pressure, travelling through the extracellular matrix (ECM), and finally entering the tumour cells and intracellular trafficking to the target.

The circulation time of a drug and drug-loaded nanoparticles in the bloodstream significantly influences its delivery to tumours. Longer circulation time increases the likelihood of the drug reaching and penetrating the tumour, particularly for targeted drug delivery systems, such as nanoparticles or antibodies, which are designed to accumulate in tumour tissues. Nanoparticles might be coated with polyethylene glycol (PEGylation) to increase the circulation time.

The tumour vasculature presents structural and functional challenges to the systemic delivery of therapeutic agents into solid tumours [6,7]. Tumour blood vessels lack the organised hierarchy in normal vasculature, displaying chaotic, dilated, and disorganised features. Abnormalities include irregular branching patterns, arteriovenous shunts, and erratic blood flow.

Tumour endothelia exhibit varying permeabilities influenced by angiogenic factors like vascular endothelial growth factor (VEGF) [8]. Additionally, vascular density is often low, since tumour cells proliferate faster than the formation of new blood vessels [3]. The size and extent of intercellular fenestra between epithelial cells varies among tissues, influencing vascular permeability. The enhanced permeability and retention (EPR) effect is based on high vascular permeability in tumours and poor lymphatic drainage that enables retention [9]. The EPR effect is essential in drug delivery but is heterogeneous within the tumour and between tumour types. The distribution of nanoparticles in tumours is notably heterogeneous, and they are often mainly located close to the capillary wall [10].

The ECM is a complex assembly of proteins and polysaccharides that provides structural support to cells and regulates critical functions such as nutrient and drug transport and waste removal [11]. This transport is governed by the ECM’s unique characteristics, like porosity and protein fibre alignment, which are influenced by the tissue’s physiological or pathological state. In tumours, the ECM exhibits altered composition and architecture, leading to a dense and stiff structure that significantly impacts the transport of molecules [12]. This modified ECM hinders the effective delivery of therapeutic agents to tumour cells and facilitates a microenvironment conducive to cancer progression and metastasis [13]. In this context, it is essential to develop delivery strategies that can enable the access and penetration of the therapeutic agents into the tumours in a localised manner.

Focused ultrasound (FUS) combined with the systemic administration of microbubbles has demonstrated an enhanced delivery of drugs and nanoparticles in preclinical studies [14,15,16]. Recent clinical trials involving patients with non-resectable pancreatic tumours treated with chemotherapy, FUS, and microbubbles reported improved patient therapeutic responses [17,18]. In preclinical and clinical studies, FUS and microbubbles have also demonstrated blood–brain barrier-opening capabilities [19,20]. Ultrasound-induced bio-effects encompass thermal and non-thermal effects. Thermal effects are considered minor in microbubble-assisted treatments with low mechanical indexes (MIs). Non-thermal effects, primarily due to acoustic radiation force and cavitation, are crucial when ultrasound is combined with microbubbles [21]. Acoustic radiation force involves momentum transfer from the ultrasound wave to tissue, causing particle and microbubble translation [22]. Cavitation, a process involving the formation and activity of bubbles, shows promising results for enhanced drug delivery. There are two types of acoustic cavitation: stable and inertial cavitation. In stable cavitation, the bubbles oscillate around an equilibrium radius over repetitive pressure cycles [23]. The oscillating bubbles can create micro-streaming in the surrounding fluid and increase the permeability of the vessel wall and cell membranes, facilitating the transport of therapeutic agents into cells [23,24]. Inertial cavitation occurs when a bubble rapidly oscillates in size, ultimately collapsing violently. The collapse of the microbubbles can create microjets and shock waves near a rigid boundary [25,26,27]. The minimum threshold required for inertial cavitation depends on the microbubbles’ initial size, gas and shell type, and the external conditions they are subjected to [21,25,26].

Acoustic Cluster Therapy (ACT^®^) presents a promising technological platform with a localised effect and enhanced drug delivery [16,28,29]. ACT^®^ is based on microclusters of microbubbles (negatively charged) and microdroplets (positively charged) forming large microbubbles under ultrasound exposure. A high-frequency ultrasound exposure initiates the activation step, causing oscillating microbubbles to transfer energy to the microdroplets, leading to an instant vaporisation of the microdroplets, forming larger ACT^®^ bubbles with a diameter of 20–22 μm [28,30]. Subsequently, a low-frequency ultrasound exposure induces the oscillations of the large microbubbles, the enhancement step, subjecting the surrounding tissue to biomechanical effects. The increased dimensions of the microbubbles employed in ACT^®^ result in an increased interaction with the vessel walls. Compared to conventional microbubbles, the larger surface area and volume of these microbubbles potentially enhance the permeability of the vascular endothelium, inducing enhanced extravasation of the co-injected drug. These microbubbles are found in a small fraction of the vessels [29] and may remain lodged for up to 10 min [28]. Previous results demonstrate that ACT^®^ can improve the therapeutic efficacy of paclitaxel and Abraxane in human prostate adenocarcinomas in mice [16], improve the therapeutic response of liposomal doxorubicin in breast tumours [31], and enhance the antitumour activity nab-paclitaxel/gemcitabine or liposomal irinotecan in a patient-derived xenograft mouse model of PDAC [32].

Intravital microscopy (IVM) enables the visualisation of biological processes in real time within the natural environment of living tissues, providing insights often unattainable through conventional microscopy and in vitro methods. IVM allows the observation of various cellular and subcellular mechanisms in tissues and organ systems [33,34]. The method’s high spatial and temporal resolution is critical for studying complex biological processes like immune responses [35,36], drug delivery dynamics [33,37], and tumour growth and metastasis [38,39]. Additionally, advancements in imaging technologies, such as multiphoton (MP) fluorescence microscopy, have significantly enhanced the capabilities of IVM [40], allowing for deeper tissue penetration and reduced phototoxicity, thus enabling longer-term studies with minimal impact on the biological systems under observation. The application of IVM in research has the potential to contribute to developing novel therapeutic strategies in areas such as oncology and immunology [35].

IVM can be applied in several animal models adapted to specific research needs. The dorsal window (DW) chamber, a notable model in cancer research, involves implanting a transparent window into the dorsal skinfold of mice or rats. This model is particularly advantageous for studying tumour growth, angiogenesis, and drug delivery dynamics due to its accessibility and ability to provide continuous imaging of the tissue area over time [34]. Another widely used model is the cranial window, which allows studying brain tissue, which is a valuable tool in neurobiology and neuro-oncology research. The chamber is surgically implanted into the skull, providing direct access to the brain while maintaining the integrity of the blood–brain barrier [41,42]. In cardiovascular research, the cremaster muscle preparation stands out for studying blood flow and leukocyte–endothelial interactions [43]. This thin muscle layer can be exteriorised while maintaining blood supply, offering clear insights into vascular dynamics. For lung studies, the intravital imaging of the lung surface provides valuable data on respiratory biology, particularly in inflammation and microcirculation studies [44]. Similarly, the mammary imaging window facilitates the study of breast cancer development and metastasis [45,46].

Performing real-time IVM during treatments such as FUS is challenging. There are a few groups that have exposed tumour tissue growing in DW chambers to ultrasound during IVM (Yemane et al. [47], Nawijn et al. [48], Zhao et al. [49]).

This study aims to develop an experimental method for studying the behaviour of ACT^®^ bubbles within the tumour’s vasculature, investigating the extravasation dynamics of co-injected macromolecules.

## 2. Materials and Methods

### 2.1. Mice

Male BALB/c Nude mice were purchased at 6–8 weeks of age (Janvier Labs, Le Genest-Saint-Isle, France) and housed individually in ventilated cages under specific pathogen-free conditions (IVCs, model 1284 L, Techniplast, Lyon, France). Mice were housed according to the recommendations of the Federation for Laboratory Animal Science Associations (FELASA). The mice also had free access to food, sterile water, and a controlled environment with a temperature of 23 °C and relative humidity between 50% and 65%, with 65 air changes per hour. For the implantation of the DW frames, the minimum weight requirement of the mice was 26 g. All experimental animal procedures complied with the protocols of the Norwegian Food Safety Authority (Mattilsynet).

### 2.2. Tumour Cell Line, Dorsal Windows (DWs), and Tumour Implantations

A human osteosarcoma cell line (OHS) was used [50] based on their abundant vascularity. The cells were cultured in Roswell Park Memorial Institute-1640 medium (Gibco Thermo-Fisher, 21875-034, Oslo, Norway), supplemented with 10% foetal bovine serum (Sigma-Aldrich, Oslo, Norway), 100 U/mL penicillin and 100 mg/mL streptomycin (Sigma-Aldrich) at 37 °C and 5% CO_2_.

**Figure 1:**
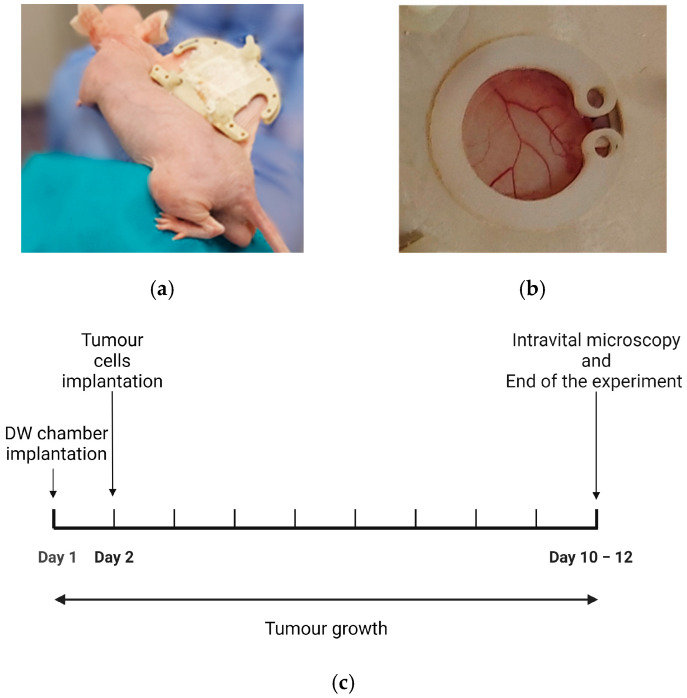
(**a**) The dorsal window (DW) chamber positioned on the back of the nude mouse. (**b**) A zoomed view of the window shows the vasculature beneath the cover glass in the tumour implanted in the chamber. (**c**) Experimental workflow: The surgical implantation of DW was performed on the 1st day, 24 h after the tumour cells were implanted on the DW; after 10 to 12 days of growth, the tumours were images by IVM.

For DW implantation, the mice were anaesthetised using a combination of fentanyl (0.05 mg/kg, Actavis Group HF, Hafnarfirdi, Iceland)/medetomidine (0.5 mg/kg, Orion Pharma, Oslo, Norway)/midazolam (5 mg/kg Accord Healthcare Limited, Devon, UK)/water in a ratio of 2:1:2:5 at a dose of 0.1 mL per 10 g body weight. Analgesia was provided through the subcutaneous injection of Metacam (5 mg/kg, 0.5 mg/mL). The surgical procedure to implant the dorsal skinfold window chamber surgery was performed as described in previous studies [34,48]. After the mice were anaesthetised, eye cream (Viscotears Liquid gel, Alcon, Fort Worth, TX, USA) was applied to keep the eyes wet, and the surgical area was scrubbed with chlorhexidine 5 mg/mL (Fresenius Kabi, Halden, Norway). The back frame of the window chamber was sutured to the back of the double layer of skin, a circular area of approximately 11 mm in diameter was cut from the skin on the front layer, and the frontal frame was attached to the back frame by puncturing the skin with an Abbocath-T catheter, 23G (Venisystems, Abbott, Sligo, Ireland) and placing the screws. The frontal part of the frame was also sutured to the skin and the back frame. A cover glass was placed on the opening, held with a ring, and the cover glass was taped to stay in place. During the implantation of the DW frames, the body temperature of the mice was maintained at a constant level using a heating pad and a heating lamp. An antidote was administered subcutaneously, consisting of atipemazol (2.5 mg/kg, Orion Pharma, Oslo, Norway), flumazenil (0.5 mg/kg, Fresenius Kabi, Bad Homburg vor der Höhe, Germany) and water (1:1:8) at a dose of 0.1 mL per 10 g. Next, the mice were placed in a recovery cabinet (Tecniplast, Philadelphia, PA, USA) with a controlled temperature of 28 °C for the post-operative needs and comfort. Figure 1a displays a mouse following surgical implantation of the DW. A zoomed-in image of the DW chamber is presented in Figure 1b.

For the implantation of the tumours, 24 h after the DW procedure, the cover glass was removed, and approximately 30 μL containing 5 × 10^6^ OHS cells were implanted in the DW chamber. The mice were anaesthetised using inhaled anaesthesia of 2–2.5% isoflurane in O_2_ and NO_2_ (Baxter, Deerfield, IL, USA). The body temperature was maintained at a constant level using a heating pad. Tumours were allowed to grow for 10 to 12 days, as shown in the experiment timeline in Figure 1c.

### 2.3. Acoustic Cluster Therapy

The ACT^®^ dispersion of microbubble–microdroplet clusters, known as PS101, was made by reconstituting a freeze-dried suspension of microbubbles of perfluorobutane (Sonazoid Powder for Injection, 16 µL/vial) with a microdroplet emulsion of perfluoromethylcyclopentane (PFMCP, 6.8 mg/mL), which was dispersed in 5 mM tris(hydroxymethyl)aminomethane (TRIS) buffer to form a 2 mL dispersion [28,29]. The microbubbles are stable thanks to a monomolecular phospholipid membrane of hydrogenated egg phosphatidylserine (H-EPS), which is negatively charged and embedded in an amorphous sucrose structure. The microdroplets are stabilised by a single layer of distearoylphosphatidylcholine (DSPC) phospholipid membrane that contains 3% (mol/mol) stearyl amine, which results in an overall positive surface charge. The PS101 dispersion consists of microbubble/microdroplet clusters with a median diameter of 4.5 μm [28]. The Sonazoid and the PFMCP emulsion were supplied by GE Healthcare AS (Oslo, Norway).

**Figure 2:**
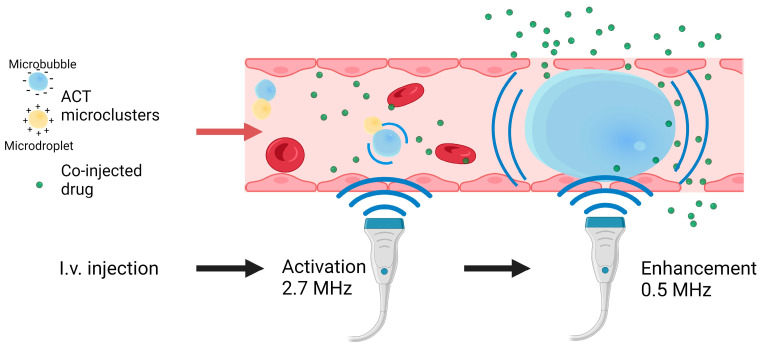
Acoustic Cluster Therapy (ACT^®^) microclusters are injected intravenously, and two ultrasound steps are applied to the tumour. The first step, activation, creates a large microbubble. The second step, enhancement, causes the large microbubble to oscillate, which leads to biomechanical effects on the vessel walls.

Prior to the administration of ACT^®^ and imaging, the mice were anaesthetised using a combination of fentanyl (0.05 mg/kg, Actavis Group HF)/medetomidine (0.5 mg/kg, Orion Pharma)/midazolam (5 mg/kg Accord Healthcare Limited)/water in a ratio of 2:1:2:5 at a dose of 0.1 mL per 10 g body weight. The ACT^®^ microbubble/microdroplet clusters were injected intravenously, and two ultrasound steps were applied: an activation step for 45 s and an enhancement step for 300 s, as represented in Figure 2. In the first step, the large microbubbles (diameter of approximately 20–30 μm) were formed. The second ultrasound step induces the oscillations of the activated microbubbles, resulting in enhanced extravasation.

### 2.4. Ultrasound Setups

A custom-made dual-frequency transducer was needed to activate the ACT^®^ bubbles and for the subsequent enhancement step. A custom-made dual-frequency transducer was employed, utilising the fifth harmonic (2.7 MHz) and fundamental (0.5 MHz) for the activation and enhancement step, respectively [51]. The transducer had a diameter of 42 mm and was placed 200 mm away from the tumour surface at an angle of 45°. The −3 dB width of the beam profile was 16 mm for a frequency of 0.5 MHz and 6 mm for a frequency of 2.7 MHz. Signals were generated using a signal generator (33500B, Agilent Technologies, Wood Dale, IL, USA) and amplified with a 50 dB RF amplifier (2100L RF Amplifier, Electronics & Innovation Ltd, Rochester, NY, USA). A switch box with two electrical circuits was employed to provide electrical matching for the two frequencies. The post-amplification signal was checked with a 10:1 attenuation probe connected from the output from the amplifier to an oscilloscope (LeCroy WaveSurfer 44Xs, LeCroy Corporation, Chestnut Ridge, NY, USA).

**Figure 3:**
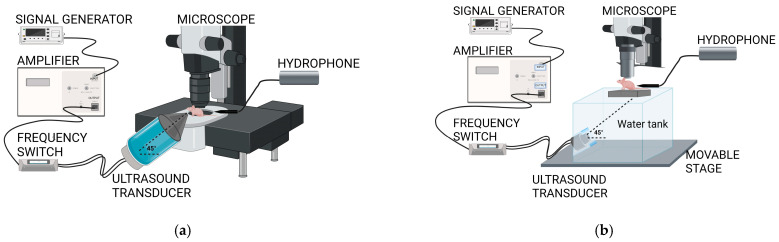
Illustration of the cone-based (**a**) and the water tank-based (**b**) ultrasound setups and the imaging setup.

To optimise the positioning of the transducer, two different setups were designed: a cone-based setup and a water-tank setup. The cone-based setup was adapted from our previous setup for imaging SonoVue microbubbles during ultrasound exposure in the DW model [47]. In the cone-based setup, the dual-frequency transducer was attached to a custom-made acrylic cone–cylinder filled with degassed water, as shown in Figure 3a. The cone–cylinder was mounted on a 3D stage to allow the movement of the transducer, which was placed at 45°. In the case of the water tank setup, the dual-frequency transducer was mounted at 45° on the side of a custom-made acrylic tank filled with degassed water (Figure 3b).

Both setups underwent acoustic characterisation with a hydrophone scanning system (Acoustic Intensity Measurement System (AIMS), III with Soniq 5.0 Software, Onda, Sunnyvale, CA, USA). The AIMS system was utilised to measure the acoustic field in water by employing a lipstick hydrophone (HGL-0200, manufactured by Onda, CA, USA). The hydrophone system provided a 2D map of the acoustic pressure at 200 mm from the transducer and the relationship between the voltage input and pressure output of the transducer. The parameters used and the expected pressures according to the characterisation are presented in Table 1.

### 2.5. Co-Alignment of the Acoustic and the Optical Field

A needle hydrophone with a 0.4 mm active element (model HNC-0400, Onda Corp., CA, USA) was employed to align the optical field of view with the ultrasound focus. The alignment was accomplished by positioning the tip of the hydrophone probe within the optical field of view and adjusting the transducer’s position until the hydrophone probe detected the peak ultrasound signal. Once the maximum signal was achieved, the position of the transducer with respect to the objective was maintained for the duration of the experiment.

### 2.6. Mouse Holder Design

**Figure 4:**
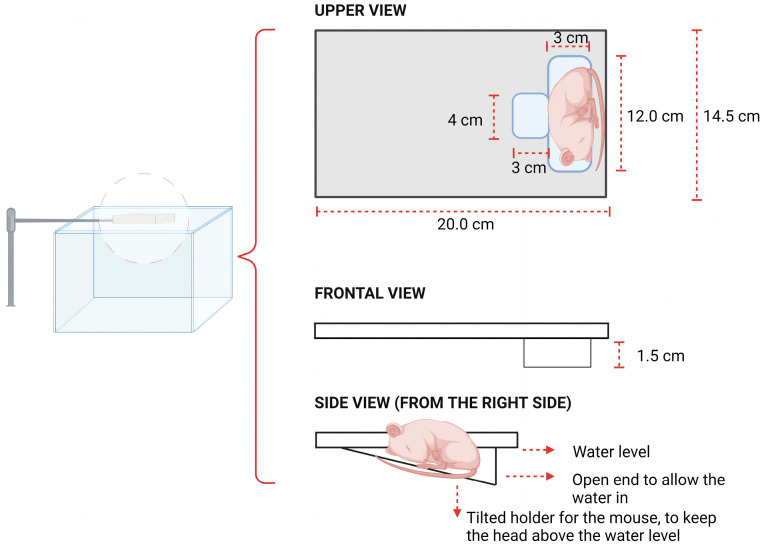
Illustration of the design of the mouse holder immersed in the water tank. The upper view shows the dimensions of the mouse bed and the dorsal window (DW) hole. The frontal view shows the thickness of the mouse holder. The side view displays the angle of the mouse bed seen from the right side of the holder and the water level.

A custom-made mouse holder was made for the mouse with the DW to be placed on the microscope (Figure 4). An independent 2D stage was adapted to the arm of the mouse holder, allowing for independent movement of the mouse and the DW chamber. The mouse was placed in the right lateral decubitus, partially submerged in the water, with the head and most of the torso above water, and the lower side of the DW chamber was submerged, thereby maintaining contact with the ultrasound beam. Two clamps held the DW chamber in place to prevent movements due to breathing that could affect the focus of the image. The metal for the holder’s material provided efficient heat transfer from the water to the animal’s body. Additionally, a rectal probe was employed to monitor the temperature continuously.

### 2.7. Multiphoton Intravital Microscopy (MP-IVM)

The imaging was performed with an upright MP microscope (Scientifica SliceScope MP Microscope, Uckfield, UK); a 20× water lens (XLUMPLFLN20 XW) with a numerical aperture of 1.0 and a working distance of 2 mm was used. Fluorescein isothiocyanate (FITC)–dextran (2 MDa) (Sigma-Aldrich) was injected intravenously (50 µL, 4 mg/mL diluted in saline) to visualise blood vessels and dextran extravasation. Two-photon excitation was carried out at 780 nm (laser: MaiTai DeepSee from SpectraPhysics, Milpitas, CA, USA), and emission detection was between 500 and 550 nm. A resonant scanner (fps = 32 f/s) or a Galvo scanner (fps = 0.76 f/s) was used. In the first case, 300 frames were acquired pre-treatment, 11,100 frames were acquired during treatment, and 100 frames were acquired post-treatment for each tumour. In the second case, 6 frames were acquired pre-treatment, 300 frames were acquired during treatment, and 6 frames were acquired post-treatment for each tumour. Each frame had a size of 512 × 512 pixels. To identify optimal imaging regions, bright field microscopy was used with the light source pE-300 LEDs (Cool-LED, Andover, UK) and the same objective as described above. The sCMOS camera (Zyla 5.5; Andor, Oxford Instruments, Abingdon, UK) was used to image the vessels. During the imaging, the mice’s temperature was regulated using a rectal probe. The injectable anaesthesia protocol applied was the same as the one stated in Section 2.2 for the DW surgical procedure. A total of 30 mice were imaged.

### 2.8. Integrating Ultrasound and Optical Imaging in Live Animal Experiments

The complexity of the experimental design lies in integrating the ultrasound system with an optical imaging system. The following steps were used to conduct the experiment:

1. Ultrasound output verification: The post-amplification voltage was verified with an oscilloscope to ensure the correct voltage levels and, thereby, the right acoustic pressure.
2. Co-alignment of acoustic and optical fields: The co-alignment was accomplished by positioning the hydrophone’s tip within the optical field of view and then adjusting the transducer’s position by moving the water tank in the XY plane through the movement of the movable stage (Figure 3b) until the hydrophone system detected the peak ultrasound signal.
3. MP microscope and sCMOS camera preparation: After alignment, the MP microscope and camera are employed to identify an optimal imaging region. Given that the position of the movable stage with the water tank cannot be changed, the 2D stage placed on the arm of the mouse holder was used to move to a different XY position in the DW chamber.
4. Mouse preparation and imaging protocol: The mouse was prepared by inserting a tail vein catheter and injecting dextran-FITC (2 MDa) to visualise the vasculature. Next, the mouse was placed on the mouse holder. Finding a region in the DW where focusing on the vessels was possible was a limiting factor. To overcome this, the strategy was to first, to find an area with a high vascular density by bright field microscopy, no bleeding, and no drainage covering the vessels. Following this, fluorescence microscopy was used to focus on vessels before optimising the focus further when the imaging mode was changed to MP microscopy.
5. Selection of the vasculature regions: To increase the probability of visualising microbubbles lodged in the vasculature, the selection of the vasculature regions to be imaged was based on two criteria: first, regions with a dense network of capillaries; and second, vessels similar in size to the activated ACT^®^ microbubbles.
6. Treatment: With the ultrasound system on, ACT^®^ microclusters were injected, and the imaging process started for the 5 min and 45 s ACT^®^ protocol. This process was repeated three times per DW: finding a new region, refocusing, re-injecting microclusters, and recording again.

### 2.9. Image Analysis

The images were analysed using ImageJ/Fiji open-source software (version 1.51j, Bethesda, MD, USA) [52]. To improve the signal-to-noise ratio, the temporal resolution of the videos was reduced by averaging the intensity of 4–10 frames, depending on the total # number of frames acquired. ROIs were drawn inside and around the vessel (free-hand selection ROI including the intravascular and the extravascular space (excluding the neighbouring vessels)), and the mean intensity was measured in all the frames to assess the intravascular and extravascular intensity over time. The intensity was presented as a relative value in relation to the background intensity of each specific acquisition by measuring the mean intensity of an ROI area with no vasculature.

#### 2.9.1. Temporal Colour Coding

The ImageJ plug-in Temporal-Color Code was used to generate a temporal-colour coded XY 2D image. This plugin allows the visualisation of changes in a series of images over time by assigning a colour to each time point in a series of grayscale images and then processing each pixel across all the images. Next, it maps the intensity values of pixels to colours based on their time point, and it creates a single composite image. The resulting image shows temporal changes as colour variations, making it possible to identify dynamic processes visually.

#### 2.9.2. Distance Map

A distance map showing the penetration of dextrans into the ECM was generated using the distance map tool in ImageJ, which creates an image where each pixel’s value represents its distance to the nearest background pixel.

#### 2.9.3. Penetration Distance

A custom-made MatLab script, adapted from the script used by Yemane et al. [47], was used to assess the penetration distance of dextrans into the ECM. The mean intensity of annuli of increasing size, each defined by two concentric circles spaced by 5 µm around the extravasation event, was calculated. Intravascular pixels were excluded. The radii of the concentric circles were successively increased from 5 to 70 µm, and the corresponding mean intensity of the annuli in each frame of the time series was obtained.

## 3. Results

### 3.1. Design of Setups

**Figure 5:**
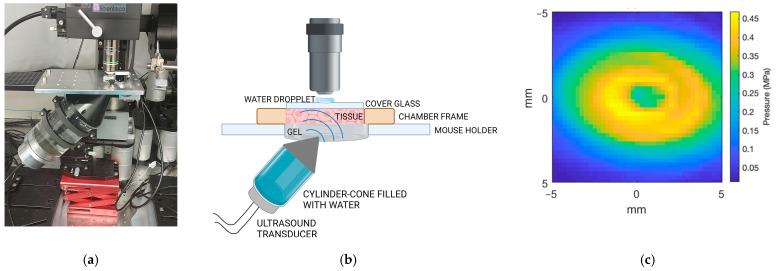
Picture of the cone-based experimental setup (**a**). Schematic of the ultrasound-imaging setup indicating the position of the transducer, the DW chamber, and the objective (not to scale) (**b**). Pressure profile from the transducer in the cone-based setup obtained by acoustic characterisation with a hydrophone scanning system (**c**).

A cone-based setup (Figure 5a), adapted from Yemane et al. [47], was initially employed for real-time imaging of ACT^®^ microbubbles and their behaviour during ultrasound exposure. In this setup, the cone–cylinder was mounted on a 3D stage and filled with water, and then ultrasound gel was applied to the tip of the cone to ensure contact between the end of the cone and the tissue at the back of the DW (Figure 5b). The cone-based setup encountered significant limitations with the dual-frequency transducer; the ultrasound field was distorted by the cone. This was characterised by a region in the centre of the ultrasound profile with low pressures (Figure 5c).

**Figure 6:**
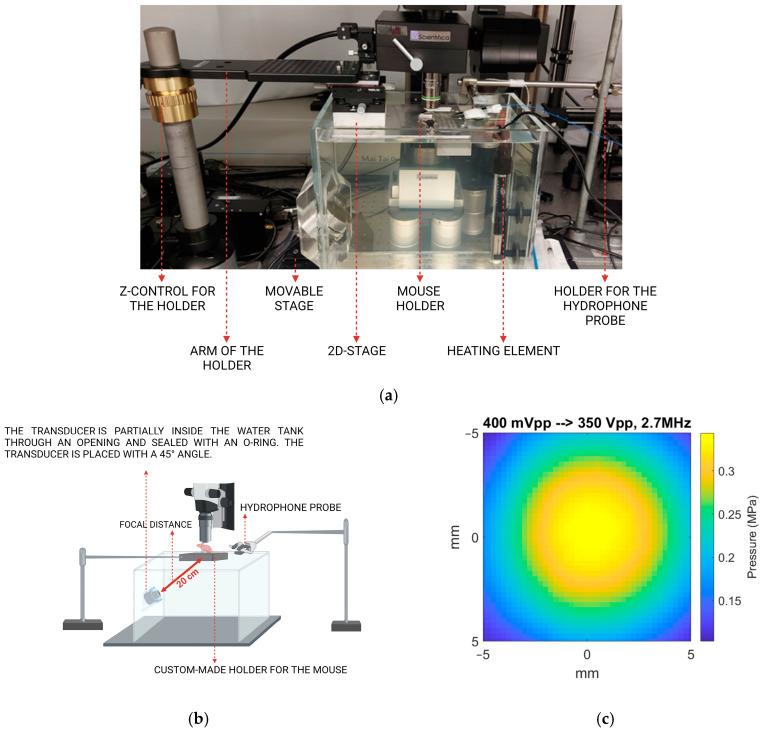
Image of the water tank-based experimental setup (**a**). Schematical representation of the design of the water tank-based ultrasound setup (**b**). Pressure profile from the transducer obtained by acoustic characterisation with a hydrophone scanning system (**c**).

Thus, the cone was removed to avoid the distortion of the ultrasound field. Figure 6a shows the experimental setup with the water tank placed on the movable stage in XY. Figure 6b shows design details. The dimensions of the water tank were designed based on three factors. First, the tank walls would be sufficiently distant from the ultrasound beam to mitigate reflections and distortion of the ultrasound profile. Second, the dimensions of the tank were determined by the physical constraints of the movable stage (under the tank), the support rail of the microscope (behind the tank), and the lens (above the tank). Third, the distance from the transducer to the DW should be 20 cm, given that this is the focal distance of the transducer. The pressure profile for the transducer in this setup is presented in Figure 6c without any distortion in the centre.

### 3.2. Real-Time Visualisation of ACT^®^ Microbubbles

**Figure 7:**
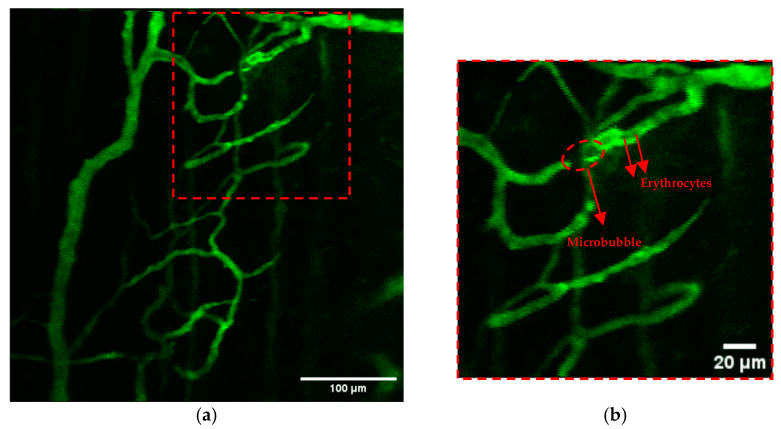
Real-time multiphoton intravital microscopy (MP-IVM) showing a microbubble and erythrocytes in the vasculature of an OHS tumour during ACT^®^ (**a**). The scale bar represents 100 μm. Zoomed-in view of the area outlined by dotted lines in panel (**a**), highlighting a microbubble and erythrocytes (**b**). The scale bar represents 20 μm.

The criteria to distinguish potentially activated microbubbles from red blood cells was based on two factors. First, their larger size, as erythrocytes have a typical diameter of approximately 7.5 to 8.7 μm, while the ACT^®^-activated microbubbles have a size distribution centred around 20–22 µm [29]. Secondly, there is a persistence of structural integrity throughout their travel in the bloodstream; in contrast, agglomerations of erythrocytes lose their apparent spherical shape as they move along the vasculature. Figure 7a shows an example of a microbubble and several erythrocytes in the same blood vessel. A zoomed-in view is displayed in Figure 7b. This microbubble had a size of 17 µm on its longest dimension and 6 µm on its shortest one. The microbubble kept the ellipsoid shape while flowing in the vessel and was lodged on a branching point, where it was temporarily stationary for 11 s. Subsequently, the ACT^®^ microbubble continued flowing downstream. An extravasation event was observed in association with the microbubble shown in Figure 7. The microbubble and associated extravasation event are shown in Supplementary Video S1.

### 3.3. Visualisation of ACT^®^-Induced Extravasation Events

**Figure 8:**
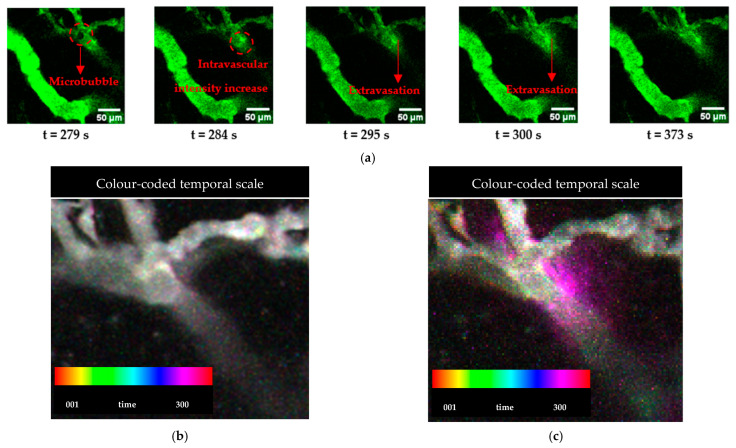
Time series of real-time MP-IVM during ACT^®^ showing a microbubble lodged at a branching point and dextran extravasation (**a**). Colour-coded temporal representation of a zoomed-in view of the extravasation area, showing the temporal behaviour of the dextran before ACT^®^ (**b**) and extravasation during treatment (**c**). Time 0 is the onset of ultrasound.

Activated ACT^®^ microbubbles and extravasation events were visualised in the same region of a vessel. Figure 8a shows a time series of an ACT^®^ microbubble lodged in the same region where dextran extravasation occurred, which was specifically upstream from where the bubble was temporarily stationary. Figure 8a shows the microbubble lodged at a branching point (t = 279 s), an increase in intravascular intensity (t = 284), subsequent extravasation (t = 295–300 s), and a moderate decrease in extravasation (t = 373 s). In this case, the temporarily stationary ACT^®^ microbubble stayed at the branching point for approximately 50 s. The microbubble had a diameter of 18 µm. Supplementary Video S2 shows the extravasation event shown in Figure 8. No extravasation events were observed before the ultrasound was applied.

### 3.4. Extravascular Accumulation Dynamics

A temporal representation of the extravasation process is shown in Figure 8b,c. This representation allows for distinguishing the dextran signal according to detection time. Figure 8b illustrates the baseline before treatment. A steady dextran signal is observed within the vascular system throughout the monitoring period. Figure 8c shows the treatment phase where a change is observed in the intensity of pixels in the extravascular area starting around 300 s. This change is consistent with when the microbubble is lodged within the vasculature.

**Figure 9:**
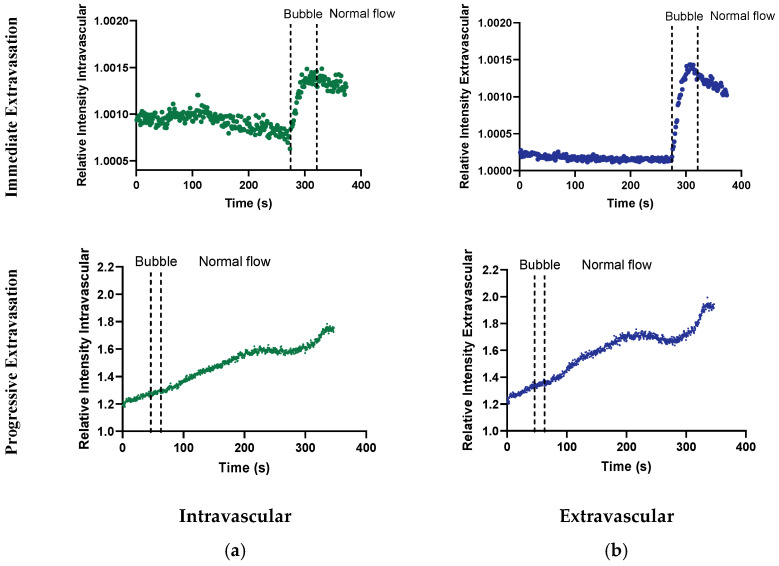
The relative mean intensity of dextran as a function of time intravascularly (**a**) and extravascularly (**b**) of two extravasation events. The figure shows when the bubble was lodged in the vasculature and when normal flow was restored. The relative mean intensity corresponds to ROIs containing the intravascular space and vicinity in the extravascular tissue. The intensity is presented relative to the background signal.

The intravascular and extravascular accumulation of dextrans were assessed by investigating the fluorescence intensity throughout the treatment in a region containing the intravascular space (Figure 9a) and the vicinity in the extravascular tissue (Figure 9b). This analysis involved two extravasation events, revealing different extravasation kinetics. Out of the total number of animals imaged, an extravasation event was observed in two mice. The first event demonstrated an immediate, fast extravasation, as indicated by the steep slope that coincides with the period when the microbubble was lodged in the vessel. The second event exhibited slower extravasation, occurring gradually after the microbubble’s lodgement within the vessel. In both cases, increased fluorescence intensity was associated with lodged microbubbles. For the fast extravasation, the bubble stayed lodged for 46 s, and the extravasation, interpreted as the time the intensity kept increasing in the extravascular region, lasted for 38 s. In the case of the slow extravasation, the bubble stayed lodged for 11 s, and the extravasation lasted for more than 282 s when the recording ended. The vessels’ diameters in these two cases were 7 and 20 µm.

### 3.5. Kinetics and Penetration of Dextrans into the Extracellular Matrix (ECM)

**Figure 10:**
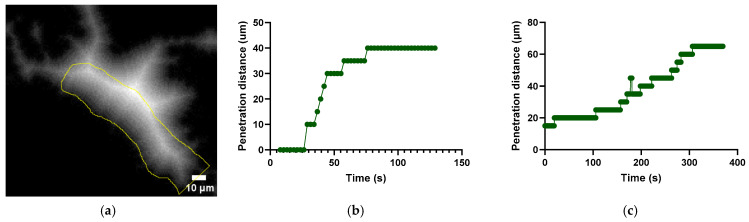
Distance map illustrating the penetration of dextran (**a**). Graph showing the penetration distance of dextran into the extravascular tissue as a function of time for the fast extravasation (**b**) and the slow extravasation (**c**).

The rate and penetration distance of dextrans into the ECM were assessed. Figure 10 shows a distance map for the fast extravasation illustrating dextran’s penetration. The graphs in Figure 10b,c show the penetration distance as a function of time during the fast and slow extravasations, respectively. These graphs corroborate the rapid and slow kinetics described in the previous section. The maximum penetration distance of the dextran was 40 and 65 µm, for the fast and slow extravasation, respectively.

## 4. Discussion

### 4.1. Experimental Setup for Visualizing ACT^®^ Microbubbles during Ultrasound

The water-tank setup allowed us to employ the dual-frequency transducer without distortion of the ultrasound beam. Characterising the acoustic pressure field was vital in ensuring a homogeneous pressure field. The custom-made mouse holder allowed direct contact between the water and the DW. Controlling the temperature is especially important during live animal imaging when the animals are anaesthetised for up to 1.5 h given that the anaesthesia impairs the normal thermoregulatory processes in mice [53].

The setup successfully integrates the MP microscope with the ultrasound setup specifically for the dual-frequency transducer. The setup allows the visualisation of ACT^®^ microbubbles and their impact on drug extravasation, affirming the potential of ACT^®^ in enhancing drug delivery within tumour tissues.

### 4.2. Visualisation of ACT^®^ Microbubbles and Extravasation Events

The criteria to distinguish activated microbubbles from red blood cells and the real-time visualisation of the bubbles are in accordance with ACT^®^ bubbles imaged in brain capillaries in the cranial window [54]. The diameter observed is consistent with the expected size distribution for these microbubbles, which is centred around 20–22 µm [29]. The observation of ACT^®^ bubbles with a diameter of approximately 18 µm lodged at vascular bifurcations in capillaries provides insights into bubble dynamics and vascular interactions.

The observed extravasation events presented two different kinetics: fast and slow. Following the microbubble’s lodging in the vessel, the fast extravasation event occurred within a few seconds and lasted for 38 s, suggesting a fast interaction between the microbubble and the vessel wall. The second extravasation event exhibited slow kinetics and lasted 282 s, suggesting a different interaction between the bubble and the vessel wall. Factors such as the diameter of the vessel and the fragility of the vessel wall could play a role in different responses. Our observations are consistent with a previous study of the blood–brain barrier where cerebral vasculature was imaged in real time through the cranial window to study the extravasation of dextran (10 kDa) [55]. Three types of extravasation kinetics were observed, all with distinct permeability constants and temporal disruption onsets: fast within a minute, sustained, which lasted for the imaging period, and slow.

ACT^®^ microbubbles facilitated dextran penetration up to 40 µm using low MIs (0.24 for activation and 0.28 for enhancement). A previous study using self-assembled nanoparticle-stabilised microbubbles or SonoVue observed comparable penetration distances of dextrans and nanoparticles [47]. For nanoparticle-stabilised microbubbles, dextran penetration ranged between 34–77 µm and 38–46 µm for different extravasation events at MIs of 0.4 and 0.2, respectively. For SonoVue, the maximum dextran penetration was 31–46 µm for different extravasation events at an MI of 0.4. The intercapillary distances in tumours are typically 100–150 μm [56]. Thus, therapeutic macromolecules penetrating 40 μm from blood vessels will reach a considerable number of tumour cells, which is important for successful cancer therapy.

The diameter of the vessels where extravasations occurred, 7 µm and 20 µm, is consistent with findings suggesting that extravasations tend to occur more frequently in smaller vessels. A computational study proposed a model to study the interaction of microbubble oscillation and compliable microvessels, predicting that decreasing the vessel size increases the circumferential stress [57].

Recent research has demonstrated the effectiveness of Antivascular Ultrasound (AVUS) using IVM in DW chambers [49]. This study employed high acoustic pressures of up to 3 MPa to induce vascular events, including vascular blood flow shutdown, occlusion, vasoconstriction, and dilation. In contrast, ACT^®^ avoids using high pressures to induce vascular effects. Acoustic peak negative pressures not exceeding 0.4 MPa are employed. A key benefit of the ACT^®^ technology is its ability to facilitate drug extravasation through gentle, low-pressure treatment, thereby reducing the risk of damage [58]. In the AVUS study, most effects were found on smaller vessels with diameters below 20 µm, especially for pressures below 2 MPa, which is consistent with our findings.

The real-time visualisation of ACT-induced extravasation events contributes to our understanding of ACT^®^ microbubble behaviour in tumour tissue and highlights the complexity of drug delivery mechanisms. The extravasation events observed are in line with the potential of ACT^®^ to enhance drug delivery to the tumour site, as demonstrated in previous preclinical studies reporting the enhanced therapeutic effect of ACT^®^ in combination with chemotherapeutic agents in several xenograft models: prostate adenocarcinoma [16], pancreatic adenocarcinoma [32], and colorectal adenocarcinoma [59]. This finding is consistent with the growing body of literature suggesting the role of ultrasound and microbubbles in enhancing drug delivery and extravasation in targeted therapies [60,61,62].

### 4.3. Limitations

The study’s main limitation is that the field of view is small, approximately 400 × 400 µm, which, combined with the low percentage of ACT^®^ microbubbles present in capillaries [29], resulted in a limited number of extravasation events reported.

Several strategies were implemented to enhance the visualisation of microbubbles and extravasation events. The selection of vessels based on size similarity to ACT^®^ microbubbles and the presence of a dense network of capillaries corresponds to previous findings that 91% of the ACT^®^ bubbles generated are <25 μm in diameter and, therefore, likely to lodge at the capillary level [29]. The preference for areas with branching points was previously found using SonoVue in the same tumour model [47]. Increasing the number of visualised tumours did not result in an increase in the visualisation of extravasation events, even though a higher pressure for activation and a higher concentration of microclusters were used, and we ultimately observed the two extravasation events reported here.

Some inter-chamber variations due to biological variability inherent in live animal experiments occurred. The clarity and cleanliness of the chambers varied between chambers, with some displaying purulent drainage, probably resulting from an immune response triggered by the implantation and the tumoural processes, which led to the accumulation of immune cells and drainage, which sometimes obscured the view of the underlying vasculature and interfered with focusing the image. The depth of the vasculature within the chambers presents another challenge for focusing. Further improvement of the DW chambers could substantially decrease the purulent drainage and the burden on the mice by reducing the weight and size of the chamber frame. A recent study has proposed 3D-printed chambers that show that light chambers reduce distress in mice and may extend the maximum dorsal skinfold chamber observation time [63].

## 5. Conclusions

We have successfully designed an experimental method for imaging ACT^®^ microbubbles and associated extravasation events by real-time MP-IVM during ultrasound treatment. As proof of concept, the microbubbles were successfully visualised, and extravasation events presenting two different kinetics were observed. Our results extend the understanding of ACT^®^ by showing the extravasation kinetics and penetration distance of therapeutic agents into the tumour interstitium. However, the number of observed extravasations was limited. Nevertheless, these results underscore the potential of ACT^®^ in enhancing drug delivery in cancer therapy.

## Figures and Tables

**Table 1:** Ultrasound parameters used on the ACT^®^ method.

Ultrasound Parameters	Activation Step	Enhancement Step
Frequency	2.7 MHz	0.5 MHz
Peak negative pressure	400 kPa	204 kPa
Mechanical index	0.24	0.28
Number of cycles	8	2

## Data Availability

Data are available upon request.

## Supplementary Materials

The following supporting information can be downloaded at: https://www.mdpi.com/article/10.3390/cells13040349/s1, the videos corresponding to the two extravasation events are presented in Videos S1 and S2.
